# DIFFERENT LEVELS OF LEISURE-TIME PHYSICAL ACTIVITY AND MENTAL HEALTH FOR OLDER ADULTS WITH DIABETES IN THE PANDEMIC

**DOI:** 10.1093/geroni/igac059.1894

**Published:** 2022-12-20

**Authors:** Jungjoo Lee, Junhyoung Kim, Sua Han

**Affiliations:** Indiana University, Blooimgton, Indiana, United States; Indiana University, Bloomington, Indiana, United States; Indiana University, Bloomington, Indiana, United States

## Abstract

This study is an initial investigation of the association of different levels of LTPA engagement with mental health (e.g., loneliness, happiness, and positive and negative affect) among older adults with diabetes during the COVID-19 pandemic. The COVID-19 has led public health researchers to improve mental health among older adults with diabetes. Leisure-time physical activity (LTPA) has been emerged to cope with mental health difficulties in the pandemic. Total 301 respondents were extracted from the Health and Retirement Study (HRS) based on the following two criteria: over 50 years old and the onset of diabetes. Multiple questionnaire items were used to assess mental health (i.e., loneliness, happiness, and positive and negative affect) for older adults with diabetes. Multivariate Analysis of Variance was utilized to investigate the relationships between the fixed variable (i.e., LTPA) and outcome variables (i.e., mental health). We categorized LTPA participation into three groups (i.e., low, mid, high) and examined mental health following a different level of LTPA participation. LTPA participation showed a significant group mean differences for loneliness, happiness, and positive affect, but not for negative affect. High-LTPA respondents presented lower loneliness) and higher happiness than Low-LTPA respondents. High-LTPA and Mid-LTPA respondents indicated higher positive affect than Low-LTPA respondents. This study provides evidence of the benefits of LTPA on mental health for older adults with diabetes. Our study supports the evidence that high-LPTA involvement can be effective in promoting mental health among older adults with diabetes in the COVID-19 era.

